# Association between neighborhood deprivation and type 2 diabetes risks among asthma patients: a nationwide population-based cohort study

**DOI:** 10.1038/s41598-025-09150-4

**Published:** 2025-07-15

**Authors:** Yuwen Wang, Yuhong Zhang, Kristina Sundquist, Jan Sundquist, Huifang Yang, Xinjun Li

**Affiliations:** 1 School of Public Health, Ningxia Medical University, Yinchuan, China; 2 Key Laboratory of Environmental Factors and Chronic Disease Control, Ningxia Medical University, Yinchuan, China; 3 Center for Primary Health Care Research, Department of Clinical Sciences, Lund University, Jan Waldenströms gata 35, 20502 Malmö, Sweden; 4 University Clinic Primary Care, Skåne University Hospital, Region Skåne, Sweden; 5 Departments of Family and Community Medicine and of Epidemiology, The University of Texas Health Science Center, Houston, TX USA; 6 Center for Community-based Healthcare Research and Education (CoHRE), Department of Functional Pathology, School of Medicine, Matsue, Shimane University, Matsue, Japan

**Keywords:** Asthma, Neighborhood deprivation, Type 2 diabetes mellitus, Sweden, Risk factors, Metabolic disorders, Psychiatric disorders

## Abstract

This study aimed to investigate the potential impact of neighborhood deprivation on the incidence of type 2 diabetes mellitus (T2DM) among individuals with asthma. This nationwide, open cohort study conducted in Sweden from 1997 to 2018 included asthma patients of all ages (n = 1,051,240) to assess their subsequent risk of developing T2DM. The study compared asthma patients residing in highly deprived neighborhoods with those living in moderately or less deprived areas (reference group). Cox proportional hazards regression models were employed to analyze the association between neighborhood deprivation and T2DM incidence, with results expressed as hazard ratios (HRs) and 95% confidence intervals (95% CIs). Analyses were stratified by sex and adjusted for potential confounders. A significant association between neighborhood deprivation and T2DM risk in asthma patients was shown. These associations remained statistically significant after adjusting for confounders, with adjusted HRs of 1.44 (95% CI 1.38–1.50) for men and 1.51 (95% CI 1.46–1.57) for women. Furthermore, a graded relationship was observed, with higher levels of neighborhood deprivation associated with an increased incidence of T2DM among asthma patients. These findings underscore critical clinical and public health concerns. The results of this study provide valuable insights for policymakers, highlighting the need to consider neighborhood deprivation when allocating resources in primary healthcare settings. They also offer guidance for optimizing clinical care strategies for patients in socioeconomically disadvantaged communities.

**Supplementary Information:**

The online version contains supplementary material available at 10.1038/s41598-025-09150-4.

## Introduction

Asthma is a chronic disease, where the inflammatory processes of the airways often result in airflow obstruction and increased bronchial hyperresponsiveness to various stimuli. Another chronic disease is type 2 diabetes mellitus (T2DM) that is a multifactorial condition primarily marked by decreased insulin sensitivity, defects in insulin secretion, and chronic inflammation. Numerous studies have indicated a significant association between asthma and an elevated risk of developing T2DM which highlights a positive correlation between the two conditions^1–4^. For instance, a study conducted on Chinese individuals in Singapore revealed that the incidence of diabetes among asthma patients increased by 31% compared to individuals with no history of asthma^2^. Another study found a relative risk of 1.37 (95% confidence interval 1.20–1.57) for T2DM among individuals with asthma compared to women without asthma^3^. Additionally, adolescents with active asthma have been shown to have a higher risk of developing T2DM^5^. The increased prevalence of T2DM in asthma patients may be attributed to several factors, including genetic predispositions, lifestyle choices, adverse effects of medication, and shared environmentally related pathophysiological processes.

Communities play a crucial role as a context for understanding health outcomes, as they possess characteristics that may influence the distribution of individual-level risk factors, such as diet and physical activity, and can interact with those individual factors. Given the pronounced residential segregation based on race and socioeconomic status, neighborhood conditions can also contribute to inequalities in disease risk. Several mechanisms within the neighborhood setting may elevate the risk of T2DM^6–8^. These include unequal access to health promotion resources, a higher prevalence of T2DM risk factors (e.g., obesity), and limited access to primary healthcare services. The incidence of T2DM is known to vary according to a person’s socioeconomic status (SES), and in addition to individual-level socioeconomic factors, neighborhood-level characteristics can further exacerbate T2DM risk. For example, research conducted in Sweden has demonstrated that an increase in neighborhood deprivation—after controlling for individual-level characteristics—is linked to a higher incidence of T2DM^9^. Moreover, refugees from areas marked by greater neighborhood poverty face a heightened risk of developing diabetes^9,10^. Furthermore, risk factors for T2DM, such as obesity and cardiovascular disease, differ significantly based on the level of neighborhood deprivation^11–13^.

Socioeconomic status and neighborhood deprivation are also associated with risk of asthma, with studies showing that children born in census tracts with higher population densities, low-income households, and higher rates of poverty, the risk ratio of asthma incidence is higher in older adults^14,15^. However, the specific relationship between neighborhood deprivation and T2DM risk in patients with asthma remains to be fully explored. Understanding this association is critical for identifying asthma patients who may be at increased risk for developing T2DM, thereby informing targeted interventions and resource allocation.

Therefore, we aimed to assess the association between neighborhood deprivation and the onset of new T2DM in patients diagnosed with asthma or receiving asthma medication in a nationwide follow-up study. Specifically, we sought to determine whether there are differences in T2DM incidence between individuals with asthma living in low-income communities compared to those residing in more affluent neighborhoods. Additionally, we intend to examine individual-level sociodemographic characteristics—such as age, country of origin, education, household income, marital status, and region of residence—as well as comorbidities including alcoholism and related liver disease, anxiety, chronic obstructive pulmonary disease (COPD), depression, hypertension, and obesity.

## Methods and materials

This study is a nationwide cohort investigation focusing on asthma patients across all age groups, with an observation period extending from January 1, 1997, to December 31, 2018. We established a baseline for each patient based on a diagnosis of asthma or the use of asthma-related medications within the observation timeframe. The primary outcome of interest was the incidence of new-onset T2DM. The main exposure variable was the level of community deprivation, which we categorized into high, moderate, and low deprivation levels; patients living in more affluent communities served as the control group. In conducting this study and drafting the manuscript, we adhered to the STROBE statement checklist to ensure methodological rigor and transparency in our cohort study.

This research was conducted at Lund University, Sweden. The data are derived from the National Register, which contains individual-level information on all individuals residing in Sweden.

### Study population

Patients of all ages diagnosed with asthma were identified from The National Patient Register and primary care data using the 10th Edition of the International Classification of Diseases (ICD-10) code J45 and J46 spanning from 1997 to 2018. Throughout the study, we identified a total of 1,058,399 individuals with asthma. Of these, we excluded 3941 individuals (0.4%) with an unknown neighborhood deprivation index and 3218 individuals (0.3%) who had a prior diagnosis of diabetes, identified by ICD-9 codes 250 between 1993 and 1996. Ultimately, a total of 1,051,240 individuals with asthma (99.3% of the original cohort) met the inclusion criteria and constituted the study population for this research (Fig. S1).

### Outcome variable

In this research, the primary diagnosis of T2DM was defined as the first recorded diagnosis in the National Patient Register, represented by the ICD-10 code E11-E14, and medication treatment with Anatomic Therapeutic Chemical classifications (ATC) code A10, during the study period.

### Main predictor variable

To characterize the neighborhood-level status for each patient, we employed a summary measure known as the Neighborhood Deprivation Index. This measure was established by identifying various deprivation indicators utilized in previous studies to define neighborhood environments^16^. We utilized small area market statistics (SAMS) as proxies for neighborhoods, as has been done in previous research^16^. SAMS represent geographic areas comprising an average of 1000–2000 residents and are delineated by homogeneous building types. All addresses of Swedish adults have been geocoded to facilitate inclusion in these small geographic units. To determine which deprivation indicators to incorporate into the calculation of each SAMS deprivation index, we conducted a principal components analysis, following the methodology outlined.

The Neighborhood Deprivation Index was specifically calculated based on residents aged 25–64 years. This age group was selected as they are generally considered to be more socioeconomically active compared to other age charities (e.g., the working population). We defined four key deprivation indicators for residents aged 25–64: a low level of education (defined as less than 10 years of formal education), a low income level (considered as total income from all sources, including interest and dividends, amounting to less than 50% of the individual median income), unemployment (not engaged in employment, excluding full-time students, individuals in mandatory military service, and early retirees), and reliance on social welfare assistance. Each of the four deprivation indicators was incorporated into the first principal component, which exhibited similar factor loadings (ranging from + 0.47 to + 0.53) and accounted for 52% of the variance among these variables. Subsequently, Z-scores were computed for each neighborhood SAMS. These Z-scores were weighted by the coefficients of the eigenvectors and summed to produce the final index. The deprivation index was classified into three categories: low deprivation (below one standard deviation from the mean), high deprivation (above one standard deviation from the mean), and moderate deprivation (within one standard deviation of the mean). Higher index scores indicate more deprived neighborhoods, while lower scores reflect more affluent (less deprived) neighborhoods. The data required for the calculation of the neighborhood deprivation variable was sourced from the Total Population Register, a total of 6143 neighborhoods were included at baseline (Supplementary Table S1).

### Covariates

All individual‐level variables were assessed on the year of asthma diagnosis and were included as they may act as confounders in the relationship between neighborhood deprivation and T2DM in patients with asthma because of their association with both the predictor and the outcome. Data on individual‐level sociodemographic factors were collected from the Total Population Register. Age was used as a continuous variable. *Educational attainment in parents* was divided into three groups based on: completion of compulsory school or less (< 9 years), practical high school or some theoretical high school (10–11 years), or theoretical high school and/or college (≥ 12 years). Parental educational level was used for children aged below 20 years. *Family income* was calculated as the sum of all family members’ incomes, multiplied by the individual family member’s consumption weight (i.e. whereby small children were given lower weights than adolescents and adults), and divided by the total consumption weight of the family members. Country of origin was categorized as ‘born in Sweden’ and ‘born outside Sweden’. Marital status was defined as ‘never married, widowed, or divorced’ or ‘married/ cohabitating’. Region of residence was categorized into ‘small towns/ rural areas’, ‘middle‐sized towns’, or ‘large cities’ (Stockholm, Gothenburg, and Malmö). *Mobility (moved)* was based on the length of time having lived in the neighbourhood, categorised as lived in neighbourhood < 5 years or ≥ 5 years. *Comorbidities* were identified from the National Patient Register during the study period and were defined as follows: hypertension (I10-I19); obesity (E65-E68); COPD (J40-J47); alcoholism (F10 and K70); depression (F32 and F33); and smoking (F17, T65.2, Z71.6, Z72.0).

### Data sources

This nationwide retrospective cohort study utilized the comprehensive Swedish national registers. The data sources included National Patient Register, containing all hospital discharge diagnoses from 1964 to 2018, with nationwide coverage from 1987, and hospital outpatient diagnoses from 2001 to 2018. Additionally, the total Population Register was used, containing data on date of death, marital status, education, migration, and other demographic variables with a high degree of coverage. The Swedish Cause of Death Register, providing cause of death data from 1961 to 2018, and the Swedish Prescribed Drug Register (2005–2018), were also utilized. Prescribed medication was classified using ATC code. Additionally, the data was obtained from a nearly nationwide collection of primary healthcare information spanning 20 out of the 21 administrative healthcare regions in Sweden, covering the period from 1990 to 2018. All data from these various sources were linked through the national 10-digit civic registration number, which is assigned to individuals at birth or upon immigrating to Sweden. To protect individual privacy, these registration numbers were substituted with pseudonymized serial numbers. The loss to follow-up was minimal, thanks to the civic registration system, and only a small fraction—less than one percent—of asthma patients were excluded due to absent SAMS codes.

Patients with missing data on other covariates were not excluded from the analysis; instead, those lacking information on family income and educational attainment were classified into the lowest income bracket (1.1% missing) and the lowest educational level (1.0% missing), respectively. For the country of origin, the four patients with missing information were categorized as being from Sweden, while the 3.5% with missing regional data were included in the “large cities” category.

### Statistics

Descriptive statistics were generated for the study population and each variable. Follow-up started from the time point of diagnosis of asthma (baseline) throughout the observation period and ended until the initial diagnosis of T2DM, death, emigration, or the end of the study on December 31, 2018. To investigate the relationships between neighborhood deprivation, various covariates, and the timing of the first diagnosis of T2DM, Cox proportional hazard models were employed. The stratified Cox proportional hazards model produced hazard ratios (HR) along with 95% confidence intervals (95% CI) for T2DM, adjusted for individual-level factors. The primary analysis was structured around three models. Model 1 utilized a univariable approach adjusted solely for age. Model 2 incorporated adjustments for age as well as individual-level sociodemographic factors. Model 3 represented a comprehensive model that included all covariates. Separate analyses were conducted for male and female participants.

A sensitivity analysis was also conducted, which accounted for patients receiving antidiabetic treatments as a surrogate measure for T2DM, defined by ATC codes (A10) obtained from the Swedish Prescription Register, covering the period from July 1, 2005, to December 31, 2018. This sensitivity analysis included all patients who had been prescribed and picked up either insulin or an oral antidiabetic medication during this time frame. We additionally examined the potential mediating role of certain comorbidities in the relationship between neighborhood deprivation (ranging from low to high) and the incidence of T2DM among asthma patients. Furthermore, we conducted an additional sensitivity analysis of the risk of diabetes in asthma patients with varying disease severities, risks of T2DM among asthma patients diagnosed in hospitalization and in primary healthcare were analyzed separately. A further adjustment was made for competing risks for type 1 diabetes as a competing risk for T2DM. The interrelationships between the variables that may affect the exposure and outcome variables were plotted in a directed acyclic graph (DAG) (Fig. S4) using the software at Dagitty.net (version 3.0)^17^ (see Supplementary Table S6 for Digital Content for the full code for reproducing our DAG at Dagitty.net). The variables’ mutual relation with each other, the exposure, and the outcome measure were considered and marked with a pointed arrow in the potential affected direction as found relevant from a clinical standpoint and the medical literature.

Moreover, interaction tests were conducted to explore whether the association between neighborhood deprivation and T2DM in asthma patients was influenced by individual-level variables; however, no significant interactions were detected (data not shown). The proportional hazard assumptions were evaluated by plotting incidence rates over time and tested based on Schoenfeld (partial) residuals (*P* = 0.5650), revealing no substantial deviations from these assumptions. All statistical analyses were executed using SAS 9.4 (SAS Institute Inc.).

## Results

Table 1 outlines the study population, which included 1,051,240 asthma patients, the number of T2DM events that occurred, and the T2DM incidence and mortality rates stratified by neighborhood deprivation. During the follow-up period (with an average follow-up of 8 years), the number of T2DM cases was 31,275 for male patients and 41,673 for female patients. It was observed that the proportion of asthma patients with T2DM increased as the level of neighborhood deprivation rose. This pattern was consistent across most subgroups. The proportion of asthma patients with T2DM was particularly higher among individuals living in highly deprived communities. Data on the population community deprivation index of asthma patients and the cumulative morbidity of T2DM are shown in Supplementary Table S1.

**Table 1:** Population and events of type 2 diabetes (T2D) among asthma patients, 1997–2018.

	Population		T2D		Rate (per 100) in neighborhood deprivation level		
	No.	%	No.	%	Low	Moderate	High
Total population	1,051,240		72,948		5.0	7.5	7.9
*Age (years)*							
< 20	468,587	44.6	4006	5.5	0.8	1.0	0.7
20–29	88,004	8.4	2383	3.3	2.0	2.6	3.6
30–39	97,785	9.3	4905	6.7	3.0	5.0	7.8
40–49	103,782	9.9	9218	12.6	5.6	9.0	13.5
50–59	94,167	9.0	14,424	19.8	11.3	15.4	20.6
60 +	198,915	18.9	38,012	52.1	15.9	19.1	22.9
*Gender*							
Males	497,950	47.4	31,275	42.9	4.7	7.0	6.5
Females	553,290	52.6	41,673	57.1	5.3	8.0	9.2
*Educational level*							
Low	597,666	56.9	28,443	39.0	2.8	5.4	5.3
Middle	152,284	14.5	22,017	30.2	11.8	14.6	16.9
High	301,290	28.7	22,488	30.8	6.1	7.4	10.3
*Family income*							
Low	244,201	23.2	15,966	21.9	4.7	6.7	7.2
Middle low	280,938	26.7	23,144	31.7	5.6	8.5	10.1
Middle high	263,252	25.0	18,684	25.6	5.3	7.7	8.1
High	262,849	25.0	15,154	20.8	4.5	6.9	5.5
*Immigrant status*							
Sweden	932,222	88.7	58,093	79.6	4.7	7.0	6.2
Other countries	119,018	11.3	14,855	20.4	9.0	12.3	14.0
*Region of residence*							
Large cities	627,212	59.7	41,225	56.5	4.8	7.3	7.4
Southern Sweden	259,047	24.6	18,408	25.2	5.1	7.3	8.7
Northern Sweden	164,981	15.7	13,315	18.3	6.0	8.4	9.7
*Mobility*							
Not moved	783,417	74.5	56,109	76.9	5.1	7.9	8.0
Moved	267,823	25.5	16,839	23.1	4.7	6.5	7.4
*Comorbidities*							
Obesity	46,592	4.4	9492	13.0	19.0	20.4	21.1
Depression	69,513	6.6	7029	9.6	7.7	10.0	12.8
Hypertension	121,565	11.6	30,766	42.2	21.5	25.1	30.1
Alcoholism	32,228	3.1	3480	4.8	8.5	11.1	12.3
Smoking	10,579	1.0	1487	2.0	11.4	13.2	16.9

Table 2 presents the hazard ratios (HRs) for T2DM incidence among asthma patients of both sexes at different levels of neighborhood deprivation. The results indicate that, compared to their peers living in moderately deprived communities (HR = 1.24, 95% CI = 1.20–1.29), male asthma patients residing in highly deprived communities had a significantly higher T2DM incidence rate (HR = 1.69, 95% CI = 1.62–1.76). Similarly, female asthma patients living in moderately deprived communities (HR = 1.33, 95% CI = 1.29–1.37) and in highly deprived communities (HR = 1.91, 95% CI = 1.84–1.98) had an increased risk of developing T2DM. Results from the full model, which adjusted for individual-level variables, showed a reduction in HRs for both male and female patients. However, HRs in the full model remained significant in moderately deprived communities (men, HR = 1.17, 95% CI = 1.13–1.21; women, HR = 1.21, 95% CI = 1.17–1.25) and highly deprived communities (men, HR = 1.44, 95% CI = 1.38–1.50; women, HR = 1.51, 95% CI = 1.46–1.57). The gender-specific analysis and data on the adjusted individual-level variable risk ratios are presented in Tables S2 and S3. The cumulative prevalence of T2DM (%) among asthma patients of different sexes, stratified by neighborhood deprivation index, showed variation. The prevalence increased with higher levels of neighborhood deprivation but declined once the deprivation index exceeded a value of 2. These findings are illustrated in Fig. S2.

**Table 2:** Association between neighborhood deprivation and T2D among asthma patients.

	Model 1			Model 2			Model 3		
	HR	95% CI		HR	95% CI		HR	95% CI	
Men									
Neighborhood deprivation (ref. Low)									
Moderate	1.24	1.20	1.29	1.19	1.15	1.23	1.17	1.13	1.21
High	1.69	1.62	1.76	1.49	1.43	1.56	1.44	1.38	1.50
Women									
Neighborhood deprivation (ref. Low)									
Moderate	1.33	1.29	1.37	1.24	1.21	1.28	1.21	1.17	1.25
High	1.91	1.84	1.98	1.62	1.56	1.68	1.51	1.46	1.57
All*									
Neighborhood deprivation (ref. Low)									
Moderate	1.29	1.26	1.32	1.23	1.20	1.26	1.20	1.17	1.23
High	1.80	1.75	1.85	1.57	1.53	1.61	1.48	1.44	1.52

Model 1: Adjusted for age; Model 2: Adjusted for age, educational level, immigrant status, region of residence, and mobility; Model 3: Fully adjusted model.

*: Gender was adjusted in model 2 and model 3.

Table 3 presents the results of a Cox regression model assessing the hazard ratios (HRs) for T2DM in all asthma patients, adjusted for individual-level variables. The association between neighborhood deprivation and T2DM events was similar to the sex-stratified analyses. Specifically, compared to low neighborhood deprivation, the fully adjusted HRs for T2DM in moderately and highly deprived neighborhoods were 1.20 (95% CI = 1.17–1.23) and 1.48 (95% CI = 1.44–1.52), respectively. In the full model, individual-level variables were significantly associated with the HR for T2DM in asthma patients. For example, the HR for T2DM was higher in women compared to men. Higher HRs were also observed in patients with a moderate level of education, lower household income, those born outside Sweden, those born in northern Sweden, and those with comorbidities.

**Table 3:** Hazards ratios (HR) and 95% confidence intervals (CI) for type 2 diabetes; Results of Cox regression models.

	Crude model				Fully adjusted			
	HR^a^	95% CI		*P*-value	HR^b^	95% CI		*P*-value
Neighbourhood deprivation (ref. Low)								
Moderate	1.29	1.26	1.32	< 0.0001	1.20	1.17	1.23	< 0.0001
High	1.80	1.75	1.85	< 0.0001	1.48	1.44	1.52	< 0.0001
Gender to males (ref. Females)	1.20	1.18	1.22	< 0.0001	1.30	1.28	1.33	< 0.0001
Age	1.04	1.04	1.04	< 0.0001	1.05	1.04	1.05	< 0.0001
Educational level (ref. High)								
Low	1.10	1.08	1.12	< 0.0001	1.25	1.22	1.27	< 0.0001
Middle	1.35	1.32	1.38	< 0.0001	1.27	1.24	1.30	< 0.0001
Family income (ref. Highest quartiles)								
Low	1.13	1.10	1.16	< 0.0001	0.92	0.90	0.95	< 0.0001
Middle-low	1.06	1.03	1.09	< 0.0001	0.93	0.90	0.95	< 0.0001
Middle-high	1.04	1.02	1.07	0.0014	0.96	0.93	0.98	0.0017
Born other countries (ref. Born in Sweden)	1.68	1.64	1.72	< 0.0001	1.60	1.56	1.64	< 0.0001
Region of residence (ref. Large cities)								
Southern Sweden	1.06	1.04	1.08	< 0.0001	1.06	1.04	1.09	< 0.0001
Northern Sweden	1.11	1.08	1.13	< 0.0001	1.16	1.13	1.19	< 0.0001
Mobility (ref. Not moved)	0.99	0.97	1.01	0.1376	0.95	0.93	0.97	< 0.0001
Comorbidities (ref. Non)								
Obesity	4.01	3.91	4.12	< 0.0001	3.78	3.68	3.88	< 0.0001
Depression	1.50	1.46	1.55	< 0.0001	1.20	1.16	1.24	< 0.0001
Anxiety	1.49	1.45	1.53	< 0.0001	1.22	1.18	1.26	< 0.0001
Smoking	1.58	1.49	1.68	< 0.0001	1.28	1.20	1.36	< 0.0001
Alcoholism	1.56	1.50	1.63	< 0.0001	1.22	1.17	1.27	< 0.0001

^a^: Crude model: adjusted for age; ^b^: Fully adjusted.

Furthermore, we conducted an additional analysis of the risk of diabetes in asthma patients with varying disease severities. In Supplementary Table S4, compared to patients living in low-poverty communities, asthma patients hospitalized in moderately deprived communities had a higher risk of developing diabetes (HR = 1.18, 95% CI = 1.13–1.23), as did those hospitalized in highly deprived communities (HR = 1.43, 95% CI = 1.36–1.50). Similarly, asthma patients diagnosed in primary healthcare settings in moderately deprived communities also had an elevated risk of diabetes (HR = 1.20, 95% CI = 1.17–1.24), as did those in highly deprived communities (HR = 1.50, 95% CI = 1.45–1.56). This increased risk was particularly pronounced in communities with higher levels of neighborhood deprivation.

Regarding other individual-level variables, age and household income did not significantly influence the risk of diabetes based on the severity of asthma. However, other individual factors did have varying effects on diabetes risk depending on the severity of the asthma condition.

In another fully adjusted sensitivity analysis (Table S5), which tracked a sub-sample of male and female asthma patients from 2006 to 2018, both neighborhood deprivation and the treatment approach for T2DM were found to be significantly associated with the incidence of T2DM in asthma patients. For instance, asthma patients with T2DM receiving inpatient care in moderately deprived communities had a lower HR (HR = 1.18; 95% CI = 1.13–1.23) compared to those receiving pharmacological treatment in the same communities (HR = 1.22; 95% CI = 1.18–1.26). Similarly, inpatient-treated patients in highly deprived communities had a lower HR (HR = 1.43; 95% CI = 1.36–1.50) than their counterparts on pharmacological treatment (HR = 1.50; 95% CI = 1.45–1.56). This association was significant across different levels of community deprivation, with the strongest effect observed in highly deprived communities.

**Fig. 1:**
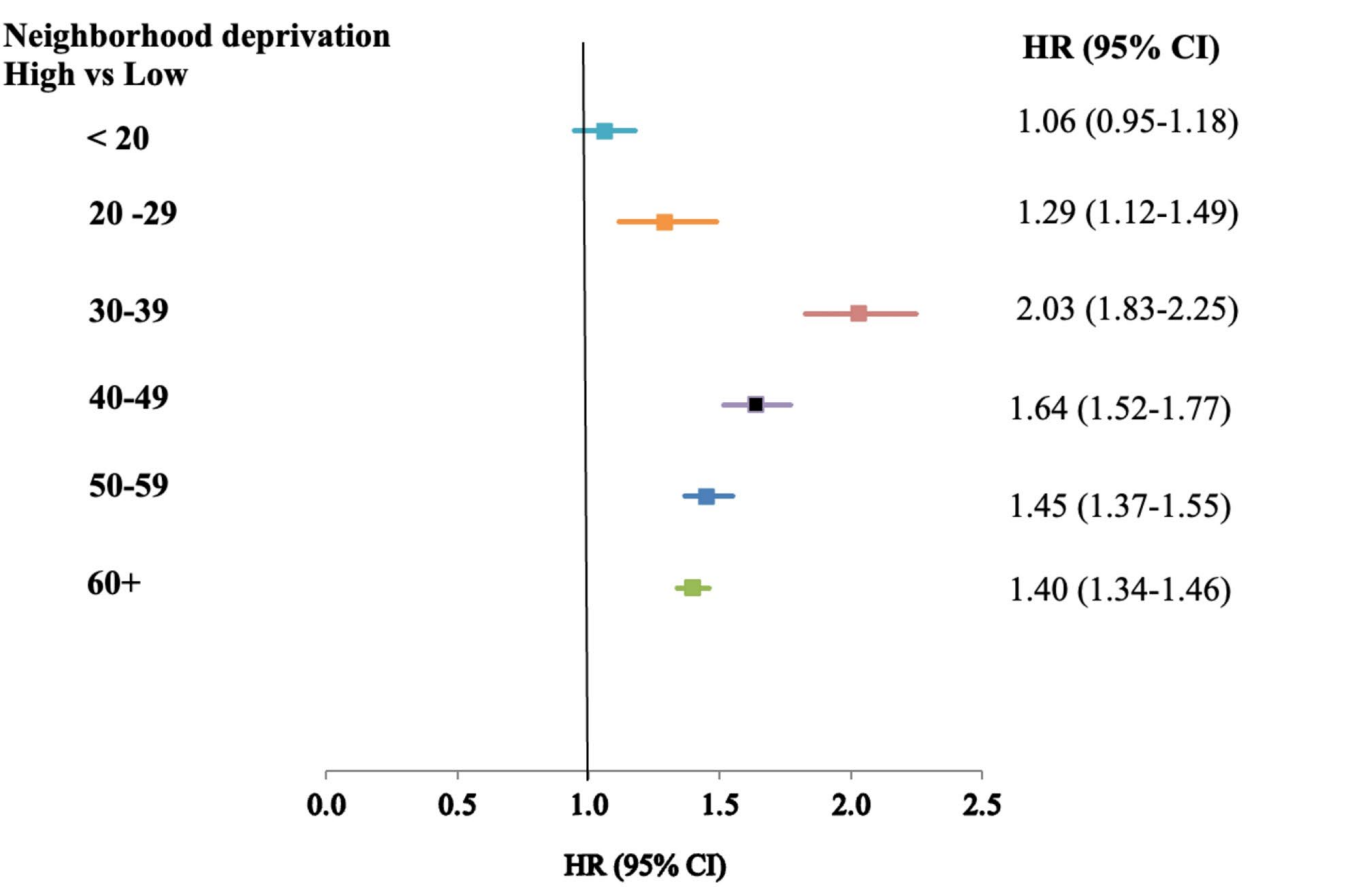
Hazards ratios (HR*) and 95% confidence intervals (CI) for T2D of asthma by age at diagnosis. *: Fully adjusted.

Figure 1 illustrates the potential impact of neighborhood deprivation on T2DM events, stratified by age at the time of asthma diagnosis. In this fully adjusted analysis, patients living in highly deprived neighborhoods had a higher risk of developing T2DM compared to those in low-deprivation areas, with the association being statistically significant in all age groups except those under 20 years of age.

Figure S3 presents Kaplan–Meier survival curves showing the time to the first T2DM event at varying levels of neighborhood deprivation. As neighborhood deprivation increased, the survival probability of patients affected by T2DM decreased, indicating poorer prognosis, and demonstrating a graded effect.

A further adjustment was made for competing risks used for type 1 diabetes as a competing risk for T2DM. Specifically, compared to low neighborhood deprivation, the fully adjusted HRs for T2DM in moderately and highly deprived neighborhoods were 1.20 (95% CI = 1.17–1.22) and 1.48 (95% CI = 1.44–1.52), respectively (data not shown). Additionally, we performed further analysis accounting for clustering effects of geographic clusters and family clusters. Specifically, compared to low neighborhood deprivation, the fully adjusted HRs for T2DM in moderately and highly deprived neighborhoods were 1.20 (95% CI = 1.17–1.22) and 1.48 (95% CI = 1.46–1.51), respectively (data not shown).

## Discussion

In this nationwide cohort study of patients with asthma, we found that those residing in highly deprived neighborhoods exhibited a significantly higher incidence and risk of developing T2DM compared to individuals living in low-deprivation neighborhoods. This difference was attenuated but remained significant after adjusting for individual-level sociodemographic variables and residential mobility. The results also remained significant when adding traditional risk factors for T2DM (e.g. obesity, alcoholism, as well as smoking). The novelty of our findings includes the use of nationwide and comprehensive data to examine the relationship between two common and chronic conditions that could be generalizable to other countries’ health care policies, especially to countries with universal health care systems.

A recent review^1^ on the association between asthma and T2DM highlighted a potential bidirectional relationship between these two chronic conditions. The use of systemic corticosteroids^18,19^ and the presence of low-grade systemic inflammation^3,18,20^ have been identified as possible underlying mechanisms driving this association. This relationship appears to contribute to poor glycemic control and exacerbated pulmonary function impairment, which collectively worsen asthma management outcomes. The current nationwide study contributes to this body of evidence by identifying neighborhood deprivation as a critical risk factor for the development of T2DM among individuals with asthma.

A systematic review concluded that diabetes and related comorbidities—such as obesity, metabolic syndrome, and lifestyle factors—are significant determinants affecting asthma management and prognosis. These conditions are associated with poor disease control, increased healthcare utilization, and reduced quality of life^21^. Therefore, several plausible mechanisms may explain our findings. Studies have demonstrated that comorbidities such as T2DM are more prevalent among individuals with asthma compared to the general population^21,22^. These comorbidities are often linked to worse asthma outcomes, leading to higher healthcare resource utilization and poorer quality of life.^21,23^ Furthermore, asthma patients exhibit a higher prevalence of physical comorbidities, which may be partially attributed to the presence of low-grade systemic inflammation as a shared underlying mechanism. Chronic low-grade inflammation has been implicated in the development of diabetes, providing a potential explanation for the increased risk in this population.^3^

Social disparities play a critical role in the prevalence of T2DM and associated impairments in glycemic regulation^24^. Extensive research has established a significant correlation between neighborhood-level deprivation and T2DM prevalence^9,25^. Moreover, prior studies have demonstrated that even after adjusting for individual-level factors, residents of impoverished communities exhibit a substantially higher prevalence of T2DM compared to those in affluent communities^9,26,27^. Additionally, individuals residing in severely deprived neighborhoods are more likely to experience elevated rates of T2D and cardiovascular risk factors, such as physical inactivity, obesity, and smoking^28^. Nevertheless, the causal pathways linking neighborhood deprivation to T2DM outcomes remain inadequately elucidated^27,29^.

Considering these factors, several potential mechanisms may explain the observed findings. First, while this study does not directly assess them, differences in knowledge, attitudes, and beliefs associated with sociocultural and demographic factors at both community and individual levels could play a pivotal role. These disparities may partially account for variations in diabetes risk factors across socioeconomic strata. For instance, communities with lower socioeconomic status often experience heightened levels of stress and psychological burden^30^. The lack of economic resources and health education may lead to an unhealthy dietary structure among residents, characterized by a deficiency in fresh fruits and vegetables and nutrient-rich foods, with a potential reliance on high-calorie, low-nutrient options^31^. Furthermore, the higher crime rates observed in impoverished communities^32^, in addition to increasing general psychosocial stress, may reduce physical activity and the likelihood of regular exercise^33^, thereby raising the risk of diabetes. This dietary pattern is closely associated with the onset of chronic diseases such as obesity, which further contributes to the risk factors for diabetes. The socio-cultural norms regarding lifestyle factors contributing to diabetes may vary across communities and have an impact on residents’ health, influencing the subsequent risk of T2DM.

Another potential mechanism of influence lies in the disparity in healthcare utilization, despite Sweden’s universal healthcare system and the seemingly higher accessibility in low-income communities^34^.These disparities may be linked to differences in individual sociodemographic factors, which can affect the likelihood of obtaining prescribed medications^35^, as well as limited access to primary care services in impoverished areas^36^. Such limitations may hinder residents from receiving adequate treatment or regular health monitoring, a critical issue for asthma patients who require ongoing medical support to manage their condition effectively.

Primary care plays a vital role in identifying mild to moderate asthma symptoms and managing them through medication and health education^37,38^. However, the lack of sustained support for asthma and diabetes management in economically disadvantaged communities often results in suboptimal disease control^39^, increasing the risk of complications such as diabetes. While hospitalizations provide short-term care, asthma management demands continuous attention and follow-up. For patients living in highly deprived areas, access to ongoing medical resources and health management after hospital discharge may be limited. This lack of continuity in care can lead to poor asthma control and, consequently, a heightened risk of developing comorbid conditions such as diabetes.

The differences in treatment approaches play a critical role in shaping health outcomes for individuals in deprived environments^40^. Medication continuity and adherence are particularly vulnerable to the economic and social limitations of these areas, increasing the risk of diabetes. Hospitalization, while effective in providing short-term relief for acute symptoms, does little to address the long-term management needs of chronic conditions. In deprived neighborhoods, this disparity is further magnified by socioeconomic factors that limit access to consistent and comprehensive treatment.

Diabetes management typically requires self-monitoring of blood glucose levels, adherence to a balanced diet, and regular physical activity. However, neighborhoods with high levels of deprivation often lack the necessary support for effective diabetes management due to low health literacy and unfavorable environmental factors^41^. In contrast, asthma management often requires episodic care to address acute exacerbations, with treatments such as corticosteroids potentially interfering with diabetes control^40^. For asthma patients in deprived areas, frequent hospitalizations and persistent symptoms may disrupt their ability to diabetes management.

Higher levels of neighborhood deprivation are often associated with environments that lack resources and social support, coupled with poor environmental conditions such as air and noise pollution. These environmental stressors can directly or indirectly affect human health. For instance, prolonged exposure to polluted environments may exacerbate asthma symptoms and increase the risk of developing diabetes^6,42,43^. Additionally, environments characterized by air pollution, limited green spaces, or a lack of recreational facilities can hinder physical activity, contributing to the rise in diabetes risk factors such as obesity^44^.

However, this study also has limitations. Neighborhood deprivation is a multidimensional socioeconomic variable, encompassing aspects such as poverty, education level, employment, social support, and environmental pollution. Accurately measuring and classifying different levels of neighborhood deprivation may face challenges, including data limitations, measurement errors, and subjective assessments. Inconsistencies in the definition and measurement of neighborhood deprivation could affect the validity of the research and the reliability of its conclusions. It may also be difficult to fully control the influence of all socioeconomic factors, such as family background, work-related stress, and social networks, which may also significantly impact the incidence of diabetes^45^. Therefore, while neighborhood deprivation is an important factor, its interaction with other socioeconomic factors is likely complex and not easily categorized or attributed. It is also important to note that certain individuals changed their place of residence and neighborhood deprivation level during the follow-up. For example, many individuals were elderly, and it could be expected that some of these individuals would change residence after retiring or becoming widowed. However, we adjusted our analyses for residential mobility, and neighborhood deprivation remained associated with significantly higher T2DM incidence in this cohort of patients with asthma. In addition, although Sweden has a national healthcare system with standardized guidelines for the diagnosis of asthma and T2DM, variations in clinical practice, patient health-seeking behavior, or access to healthcare services may still lead to delayed or missed diagnoses in certain areas and/or populations. This may be particularly relevant for individuals with complex comorbidities such as obesity or hypertension, which are closely associated with T2DM and may influence diagnostic prioritization.

This study offers several strengths that help address its limitations. First, as a nationwide cohort study, it encompasses nearly all asthma patients across all age groups residing in Sweden during the study period, thereby greatly enhancing the generalizability of the findings. Moreover, the National Patient Register exhibits excellent data completeness (> 99%), and the positive predictive value for diabetes-related hospitalization data is nearly 99%^46^ensuring robust and reliable data to support the study’s conclusions. Additionally, the outcome data in this study were based on clinically recorded diagnoses by physicians rather than self-reported information, effectively eliminating recall bias. Another significant strength is Sweden’s system of assigning each resident a unique personal identification number, which was replaced with pseudonymized serial numbers for this study. This allowed the tracking of the entire study population without significant loss to follow-up. Furthermore, the study utilized highly comprehensive registries and nationwide databases^47^, with fewer than 1% of asthma patients excluded due to missing SAMS codes. In addition to its relevance for the Swedish population, the study’s use of nationwide data, high statistical power, and consistent results across subgroups suggest that the findings may be generalizable to countries with similar healthcare systems and demographic profiles, particularly to other Nordic countries.

By substituting unique personal identification numbers with pseudonymized serial numbers, the study was able to link individual-level clinical data with national demographic and socioeconomic data as well as small neighborhood units (SAMS). These small neighborhood units, consisting of relatively homogeneous building types and typically housing approximately 1000–2000 individuals, represent another key strength of the study^48^. Previous research has shown that such small-scale community units align closely with how residents perceive their neighborhoods^49^, further enhancing the precision and representativeness of the study. Lastly, the findings were in line with previous findings on neighborhood deprivation and health in general, including previous evidence on the association risk factors between neighborhood deprivation in relation to asthma^39,50^,and T2DM^9,29,51^.

## Conclusion

Neighborhood deprivation, as a critical social determinant of health, plays a significant role in influencing both asthma and diabetes. Stratifying asthma patients by levels of neighborhood deprivation provides a clearer understanding of the long-term impact of social environmental factors on the development and progression of chronic diseases such as asthma and diabetes. Investigating its dual impact offers valuable insights for advancing both health equity and the prevention and management of these conditions. Interventions such as improving community environments, enhancing health education, and ensuring equitable allocation of healthcare resources may contribute positively to reducing the prevalence of diabetes among asthma patients.

## Electronic supplementary material

Below is the link to the electronic supplementary material.

Supplementary Material 1

## Data Availability

This study made use of several national registers and owing to ethical concerns, data cannot be made openly available, and we are not allowed to share our data. Further information regarding the health registries is available from the Swedish National Board of Health and Welfare: https://www.socialstyrelsen.se/en/statistics-and-data/registers/ and Kristina Sundquist, co-author of this study and the one that holds the ethical permission for this study.
