# Supplementary material for: Association between neighborhood deprivation and type 2 diabetes risks among asthma patients: a nationwide population-based cohort study
Source: Sci Rep. 2025 Jul 15;15:25570. doi: 10.1038/s41598-025-09150-4 (PMC12264147; doi:10.1038/s41598-025-09150-4)

Supplementary materials

Table S1. Study population (asthma patients) and neighborhoods
Table S2. Association between neighborhood deprivation and T2D among asthma patients in men
Table S3. Association between neighborhood deprivation and T2D among asthma patients in women
Table S4. Hazards ratios (HR) and 95% confidence intervals (CI) for diabetes of asthma patients diagnosed in hospitalization and in primary health care; Results of Cox regression models
Table S5. Hazards ratios (HR) and 95% confidence intervals (CI) for type 2 diabetes of asthma patients, 2006-2018 (N=755099); Results of Cox regression models
Table S6. The full code of Directed acyclic graph
Figure S1. Flow-chart of study population
Figure S2. Incident rate of T2D (%) among asthma patients by neighborhood deprivation index.
Figure S3. Kaplan-Meier survival estimates of T2D among asthma patients.
Figure S4. Directed acyclic graph.

Table S1. Study population (asthma patients) and neighborhoods deprivation index

	Low	Moderate	High	Total
Number of neighborhoods (%)	1606 (26.1)	3420 (55.7)	1117 (18.2)	6143
SES index	<-1	-1-1	>1	
Study population (%)	272711 (25.9)	548986 (52.2)	229543 (21.8)	1051240
Events of T2D (%)	13622 (18.7)	41239 (56.5)	18087 (24.8)	72948

Table S2. Association between neighborhood deprivation and T2D among asthma patients in men

	Model 1			Model 2			Model 3		
	HR	95% CI		HR	95% CI		HR	95% CI	
**Neighborhood deprivation (ref. Low)**									
Moderate	1.24	1.20	1.29	1.19	1.15	1.23	1.17	1.13	1.21
High	1.69	1.62	1.76	1.49	1.43	1.56	1.44	1.38	1.50
**Age (years)**	1.05	1.05	1.05	1.05	1.05	1.05	1.05	1.05	1.05
**Educational level (ref. High)**									
Low				1.29	1.25	1.33	1.25	1.21	1.30
Middle				1.38	1.34	1.43	1.32	1.28	1.37
**Family income (ref. Highest quartiles)**									
Low				0.82	0.79	0.86	0.82	0.78	0.85
Middle-low				0.90	0.86	0.93	0.88	0.85	0.92
Middle-high				0.92	0.88	0.95	0.90	0.87	0.94
**Born other countries (ref. Born in Sweden)**				1.65	1.59	1.71	1.62	1.57	1.68
**Region of residence (ref. Large cities)**									
Southern Sweden				1.02	0.99	1.06	1.03	1.00	1.06
Northern Sweden				1.14	1.10	1.18	1.16	1.12	1.21
**Mobility (ref. Moved)**				0.98	0.95	1.01	0.95	0.92	0.98
**Comorbidities (ref. Non)**									
Obesity							3.90	3.73	4.08
Depression							1.24	1.17	1.31
Anxiety							1.23	1.17	1.30
Smoking							1.36	1.23	1.50
Alcoholism							1.30	1.23	1.38

Model 1: Adjusted for age; Model 2: Adjusted for age, educational level, immigrant status, region of residence, and mobility; Model 3: Fully adjusted model.

Table S3. Association between neighborhood deprivation and T2D among asthma patients in women

	Model 1			Model 2			Model 3		
	HR	95% CI		HR	95% CI		HR	95% CI	
**Neighborhood deprivation (ref. Low)**									
Moderate	1.33	1.29	1.37	1.24	1.21	1.28	1.21	1.17	1.25
High	1.91	1.84	1.98	1.62	1.56	1.68	1.51	1.46	1.57
**Age (years)**	1.04	1.04	1.04	1.04	1.04	1.04	1.04	1.04	1.04
**Educational level (ref. High)**									
Low				1.28	1.24	1.32	1.26	1.22	1.29
Middle				1.31	1.27	1.35	1.26	1.22	1.30
**Family income (ref. Highest quartiles)**									
Low				1.09	1.05	1.13	1.06	1.02	1.10
Middle-low				1.06	1.02	1.10	1.03	0.99	1.07
Middle-high				1.07	1.03	1.12	1.05	1.01	1.09
**Born other countries (ref. Born in Sweden)**				1.62	1.57	1.67	1.58	1.53	1.62
**Region of residence (ref. Large cities)**									
Southern Sweden				1.09	1.06	1.12	1.09	1.06	1.12
Northern Sweden				1.13	1.09	1.17	1.15	1.11	1.18
**Mobility (ref. Moved)**				0.96	0.94	0.99	0.95	0.92	0.97
**Comorbidities (ref. Non)**									
Obesity							3.63	3.51	3.75
Depression							1.17	1.12	1.22
Anxiety							1.19	1.15	1.24
Smoking							1.22	1.13	1.32
Alcoholism							1.08	1.01	1.15

Model 1: Adjusted for age; Model 2: Adjusted for age, educational level, immigrant status, region of residence, and mobility; Model 3: Fully adjusted model.

Table S4. Hazards ratios (HR) and 95% confidence intervals (CI) for diabetes of asthma patients diagnosed in hospitalization and in primary health care; Results of Cox regression models

	Hospitalization of asthma (N=499558)				Asthma diagnosed in primary health care (N=551682)			
	HR*	95% CI		P-value	HR*	95% CI		P-value
**Neighbourhood deprivation (ref. Low)**								
Moderate	1.18	1.13	1.23	<.0001	1.20	1.17	1.24	<.0001
High	1.43	1.36	1.50	<.0001	1.50	1.45	1.56	<.0001
**Age**	1.05	1.05	1.05	<.0001	1.04	1.04	1.04	<.0001
**Gender to males (ref. Females)**	1.24	1.21	1.28	<.0001	1.35	1.32	1.38	<.0001
**Educational level (ref. High)**								
Low	1.19	1.15	1.24	<.0001	1.29	1.25	1.32	<.0001
Middle	1.20	1.16	1.25	<.0001	1.31	1.28	1.35	<.0001
**Family income (ref. Highest quartiles)**								
Low	0.94	0.90	0.99	0.013	0.94	0.91	0.97	<.0001
Middle-low	0.97	0.92	1.02	0.183	0.92	0.89	0.95	<.0001
Middle-high	1.00	0.95	1.05	0.943	0.95	0.92	0.98	0.001
**Born other countries (ref. Born in Sweden)**	1.63	1.57	1.70	<.0001	1.57	1.52	1.61	<.0001
**Region of residence (ref. Large cities)**								
Southern Sweden	1.10	1.07	1.14	<.0001	1.07	1.04	1.10	<.0001
Northern Sweden	1.16	1.12	1.21	<.0001	1.17	1.14	1.21	<.0001
**Mobility (ref. Moved)**	0.99	0.96	1.02	0.551	0.93	0.91	0.96	<.0001
**Comorbidities (ref. Non)**								
Obesity	3.77	3.62	3.94	<.0001	3.79	3.66	3.92	<.0001
Depression	1.22	1.16	1.29	<.0001	1.18	1.13	1.23	<.0001
Anxiety	1.23	1.17	1.29	<.0001	1.21	1.16	1.26	<.0001
Smoking	1.32	1.20	1.45	<.0001	1.24	1.15	1.35	<.0001
Alcoholism	1.25	1.17	1.34	<.0001	1.19	1.13	1.25	<.0001

HR: Hazard ratio; CI: Confidence interval.

*: Fully adjusted.

Table S5. Hazards ratios (HR) and 95% confidence intervals (CI) for type 2 diabetes of asthma patients, 2006-2018 (N=755099); Results of Cox regression models

	Hospitalization of T2D				Medication of T2D			
	HR*	95% CI		P-value	HR*	95% CI		P-value
**Neighbourhood deprivation (ref. Low)**								
Moderate	1.18	1.13	1.23	<.0001	1.22	1.18	1.26	<.0001
High	1.43	1.36	1.50	<.0001	1.50	1.45	1.56	<.0001
**Age**	1.06	1.05	1.06	<.0001	1.04	1.04	1.04	<.0001
**Gender to males (ref. Females)**	1.50	1.45	1.55	<.0001	1.34	1.31	1.38	<.0001
**Educational level (ref. High)**								
Low	1.32	1.26	1.37	<.0001	1.26	1.23	1.30	<.0001
Middle	1.36	1.31	1.42	<.0001	1.34	1.30	1.38	<.0001
**Family income (ref. Highest quartiles)**								
Low	1.10	1.05	1.16	0.000	1.02	0.99	1.06	0.228
Middle-low	1.02	0.97	1.06	0.532	0.96	0.93	1.00	0.029
Middle-high	1.00	0.95	1.05	0.947	0.98	0.95	1.01	0.232
**Born other countries (ref. Born in Sweden)**	1.56	1.50	1.62	<.0001	1.62	1.57	1.67	<.0001
**Region of residence (ref. Large cities)**								
Southern Sweden	1.14	1.09	1.18	<.0001	1.11	1.08	1.14	<.0001
Northern Sweden	1.23	1.18	1.29	<.0001	1.21	1.17	1.25	<.0001
**Mobility (ref. Moved)**	0.89	0.86	0.93	<.0001	0.93	0.91	0.96	<.0001
**Comorbidities (ref. Non)**								
Obesity	4.26	4.06	4.47	<.0001	3.72	3.59	3.86	<.0001
Depression	1.19	1.12	1.27	<.0001	1.18	1.13	1.24	<.0001
Anxiety	1.29	1.22	1.36	<.0001	1.22	1.17	1.27	<.0001
Smoking	1.45	1.31	1.60	<.0001	1.33	1.23	1.44	<.0001
Alcoholism	1.41	1.31	1.52	<.0001	1.17	1.11	1.24	<.0001

HR: Hazard ratio; CI: Confidence interval.

*: Fully adjusted.

Table S6. The full code of Directed acyclic graph

```
dag {
bb="-10.787,-10.72,8.908,10.175"
"Educational level" [pos="-9.578,-1.556"]
"Family income" [pos="-8.932,1.574"]
"Immigration status" [pos="-0.369,-8.086"]
"Marital status" [pos="-4.066,-8.176"]
"Neighborhood deprivation" [exposure,pos="-9.261,-9.752"]
"Region of recidence" [pos="7.905,-9.707"]
Age [pos="2.572,-7.680"]
Alcoholism [pos="-6.183,7.248"]
Depression [pos="-7.543,5.672"]
Gender [pos="6.448,2.925"]
Hypertension [pos="-5.125,8.666"]
Mobility [pos="-8.354,3.938"]
Obesity [pos="-2.967,9.319"]
Smoking [pos="6.984,-0.655"]
T2D [outcome,pos="7.808,9.072"]
"Educational level" -> "Family income"
"Educational level" -> "Marital status"
"Educational level" -> "Neighborhood deprivation"
"Educational level" -> T2D
"Family income" -> "Neighborhood deprivation"
"Family income" -> T2D
"Immigration status" -> "Educational level"
"Immigration status" -> "Family income"
"Immigration status" -> "Neighborhood deprivation"
"Immigration status" -> "Region of recidence"
"Immigration status" -> Mobility
"Immigration status" -> T2D
"Marital status" -> "Family income"
"Marital status" -> Alcoholism
"Marital status" -> Depression
"Marital status" -> Hypertension
"Marital status" -> Mobility
"Marital status" -> Obesity
"Marital status" -> Smoking
"Marital status" -> T2D
"Marital status" <-> "Neighborhood deprivation"
"Neighborhood deprivation" -> Alcoholism
"Neighborhood deprivation" -> Depression
"Neighborhood deprivation" -> Hypertension
"Neighborhood deprivation" -> Obesity
"Neighborhood deprivation" -> Smoking
"Neighborhood deprivation" -> T2D
"Region of recidence" -> "Neighborhood deprivation"
"Region of recidence" -> Alcoholism
"Region of recidence" -> Depression
"Region of recidence" -> Hypertension
```

```
"Region of recidence" -> Mobility
"Region of recidence" -> Smoking
"Region of recidence" -> T2D
Age -> Alcoholism
Age -> Depression
Age -> Hypertension
Age -> Smoking
Age -> T2D
Alcoholism -> T2D
Depression -> T2D
Gender -> Alcoholism
Gender -> Depression
Gender -> Hypertension
Gender -> Smoking
Gender -> T2D
Hypertension -> T2D
Mobility -> "Neighborhood deprivation"
Mobility -> T2D
Obesity -> T2D
Smoking -> Alcoholism
Smoking -> Depression
Smoking -> Hypertension
Smoking -> Obesity
Smoking -> T2D
}
```

Figure S1. **Flow-chart of study population**

Study population	
1058399	Asthma patients diagnosed in in-patient register, out-patient register, and primary health care 1997-2018
1054458	Excluding with unknown neighborhood deprivation index (n=3941)
1051240	Excluding previous diagnosis of diabetes, 1993-1996 with ICD-9 codes 250, (n=3218)

Figure S2. Incident rate of T2D (%) among asthma patients by neighborhood deprivation index

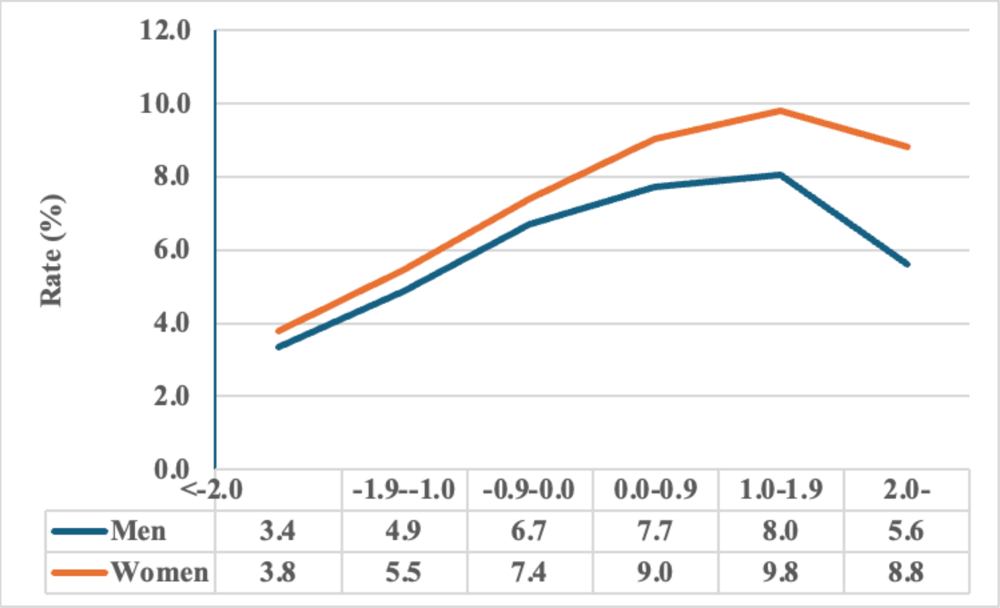

Figure S3. Kaplan-Meier survival estimates of T2D among asthma patients

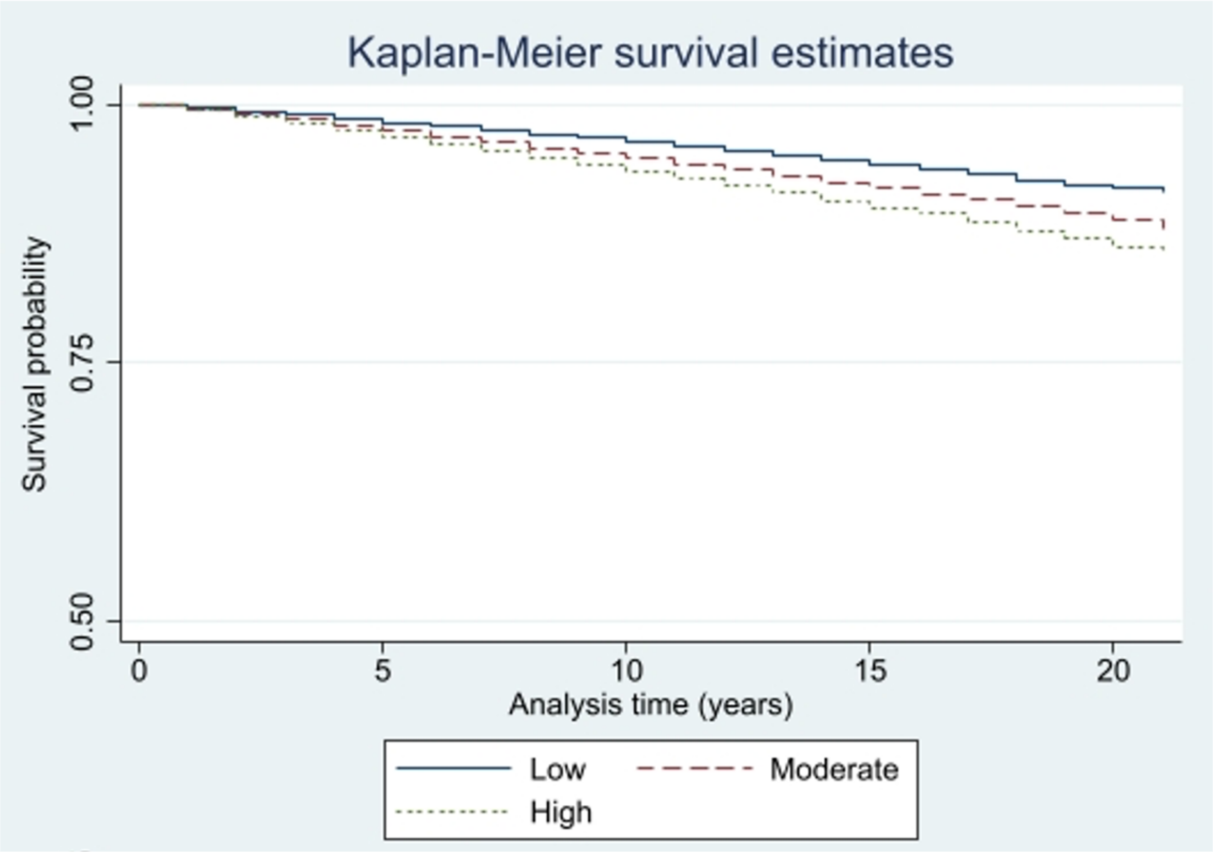

Figure S4. Directed acyclic graph.
This model was constructed at www.dagitty.net (v3.0) to obtain a minimal sufficient adjustment set for the multiple regression model. Yellow circle for exposure, blue circle for outcome, pink circle for ancestor of exposure and outcome, pink arrow for biasing path, and green arrow for causal path.

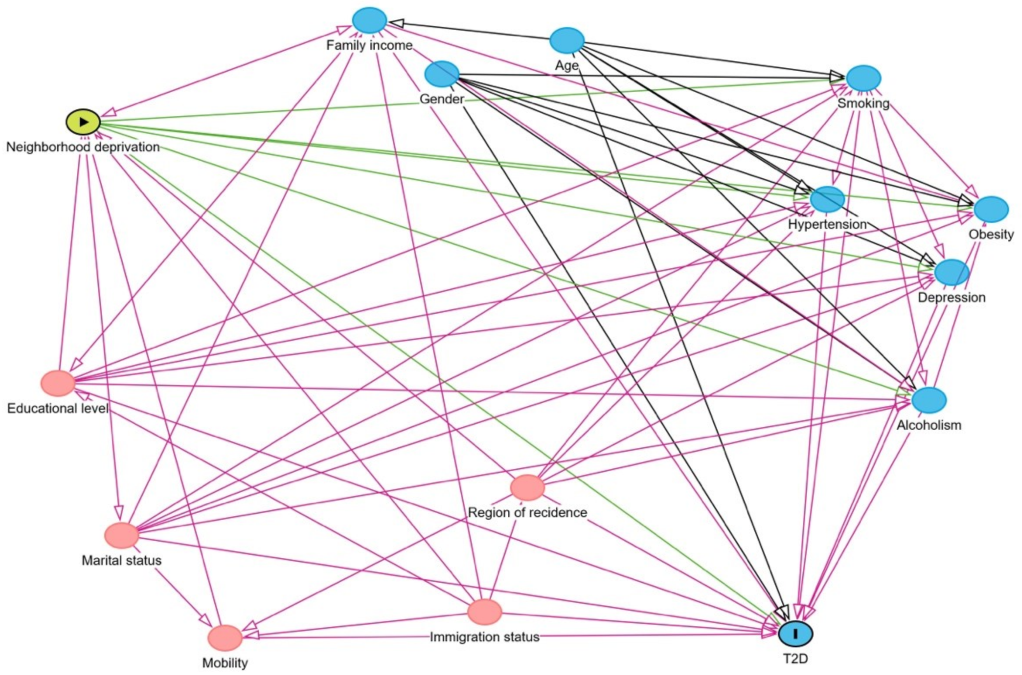

Supplement: Supplementary file 1 — Supplementary Material 1 [file 41598_2025_9150_MOESM1_ESM.pdf]
